# Effects of Substitution of Corn with *Macadamia integrifolia* Husk on Rumen Fermentation Characteristics and Microbial Flora in Goats: An In Vitro Experiment

**DOI:** 10.3390/ani16111729

**Published:** 2026-06-04

**Authors:** Xingyu Chen, Jiancheng Han, Chen Wei, Xiaoyan Deng, Kaibin Chen, Anmiao Chen, Yuanting Yang, Shiyang Huang, Xiaosong Zhang, Hui Zeng, Qian Yang, Hu Liu, Hanlin Zhou

**Affiliations:** 1 Zhanjiang Experimental Station, Chinese Academy of Tropical Agricultural Sciences, Zhanjiang 524013, China; xingyuchen2026@163.com (X.C.);; 2 College of Coastal Agricultural Sciences, Guangdong Ocean University, Zhanjiang 524013, China; 3 Institute of Agricultural and Animal Husbandry Industry Development, College of Animal Science and Technology, Guangxi University, Nanning 530004, China; 4 College of Veterinary Medicine, China Agricultural University, Beijing 100193, China; 5 Key Laboratory of Hainan Province for Postharvest Physiology and Technology of Tropical Horticultural Products, South Subtropical Crops Research Institute, Chinese Academy of Tropical Agricultural Sciences, Zhanjiang 524091, China

**Keywords:** *Macadamia integrifolia* husk, replace corn levels, in vitro, rumen fermentation, goats

## Abstract

This study was conducted to measure *Macadamia integrifolia* husk (MIH) as a feed ingredient for goats using an in vitro rumen fermentation system. Four isonitrogenous and isoenergetic substrates were prepared by using MIH to replace 0, 5%, 10%, or 15% of corn as a dry matter basis, namely MIH0, MIH5, MIH10, and MIH15 groups, respectively. Gas production decreased linearly as corn was replaced by increasing levels of MIH. The DMD decreased linearly at the 24 h and 48 h incubation times as corn was replaced by increasing levels of MIH (*p* < 0.01). The NDFD decreased linearly at the 6 h, 12 h, 24 h, and 48 h incubation times as corn was replaced by increasing levels of MIH. At 48 h, pH differed extremely significantly (*p* < 0.001) and followed a quadratic trend (*p* < 0.05). The MIH0 group had the highest NH_3_–N, butyric, and isovaleric acids concentration (*p* < 0.05); the MIH5 group had the highest MCP, acetic acid, and propionic acid concentration (*p* < 0.05). Bacteroidota and Pseudomonadota were the dominant phyla in the fermentation fluid at the 48 h incubation timepoint among the four groups. The relative abundance of Kiritimatiellota and Synergistota was highest in the MIH15 group. The relative abundance of Pseudomonadota was highest in the MIH5 group. At the genus level, *norank_p_Bacteroidota* and *Ruminobacter* were the dominant genera, and the bacterial community structure differed significantly among the four treatments (*p* < 0.05). In conclusion, we suggest that the MIH replacing 5% of corn did not affect fermentation, VFA profile, or microbial protein synthesis and it could alter the bacterial communities. Moreover, it is necessary to conduct an in vivo study to investigate the growth performance of goats in a further study.

## Simple Summary

The *Macadamia integrifolia* husk (MIH) is a potential ingredient for livestock, yet little information has been gathered exploring the replacement of corn in goats’ diets. This study used in vitro technology to measure the substitution of corn with MIH. The results showed that MIH replacing 5% of corn did not affect in vitro nutrition digestibility, VFA profile, or microbial protein synthesis and could alter the bacterial communities. In addition, it is necessary to conduct an in vivo study to investigate the growth performance of goats in a further study.

## 1. Introduction

The *Macadamia integrifolia*, commonly regarded as the “king of nuts” [1], belongs to the family Proteaceae. It was native to the subtropical rainforests of Queensland and New South Wales in Australia, and its primary production areas included South Africa, Australia, Kenya, and Hawaii [2,3]. Macadamia nuts possess extremely high nutritional value [4] and are rich in various unsaturated fatty acids beneficial to human health [5,6]. In recent years, it was introduced to regions in China such as Guangdong, Guangxi, and Yunnan. As of the end of 2022, China’s macadamia nut cultivation area accounted for approximately 60% of the global total yield, representing a significant proportion worldwide and making China the world’s largest macadamia nut-growing region [7].

The processing of *Macadamia integrifolia* generates byproducts, such as husks, which are abundant in vitamin E, phenolic acids, flavonoids, and proanthocyanidins [8]. These compounds have been reported to reduce the risk of cardiovascular and degenerative diseases [8]. Moreover, dietary supplementation with flavonoid extract could enhance lactation performance and milk quality in dairy cows by improving their inner gastrointestinal environment and health status [9]. A combined treatment consisting of *Asparagopsis taxiformis* and phloroglucinol can prevent the accumulation of H_2_ in the rumen, promote an increase in acetate production, and improve rumen fermentation [10]. However, only a small portion of these husks is used for natural fermentation in organic fertilizers, and the majority of these husks are landfilled or incinerated, resulting in low utilization efficiency. Considering that *Macadamia integrifolia* husks (MIH) are abundant, low-cost, and have potential feeding value, they warrant further research as a feed ingredient.

Corn (*Zea mays* L.) is one of the world’s three major staple crops and serves as a critical component of livestock and poultry feed, often combined with soybean meal to produce corn–soybean and wheat–corn–soybean compound feeds [11]. In recent years, intensive production has intensified competition between humans and animals for grain resources [12,13]. To alleviate competition between feed and food, the development of non-cereal feed ingredients has become a pressing priority. Notably, significant progress has been made in replacing corn kernels with corn protein feed [14]. It has been reported that agricultural and industrial byproducts have the potential to replace traditional feeds, alleviate competition between feed and food, and enhance value-added potential [15]. Based on a previous in vitro study, it was reported that adding 14–21% nut husks (almond, hazelnut, or pistachio husks, all of which contain phenolic compounds, tannin) to the diet has a minimal effect on goat digestion and does not cause adverse effects [16]. Furthermore, incorporating hazelnut husks and extruded flaxseed into lamb diets has been reported as an effective strategy to prevent lipid peroxidation, improve oxidative stability, and enhance meat quality [17]. A previous in vitro study reported that substituting different proportions of corn with dried persimmon peel, which contains tannins, flavonoids, and carotenoids, could decrease gas production, methane production, and dry matter digestibility [18]. An in vivo study reported that replacing peanut straw with 5% ginger straw, which contains curcumin, gingerol, and flavonoids, in the goat diet resulted in higher NDF digestibility and growth performance [19]. Other findings suggested that replacing 7.88% of peanut vines with Chrysanthemum stalks, which contain terpenoids, flavonoids, polyphenols, and polysaccharides on a dry matter basis, yielded the most effective result in Hu sheep [20].

To date, the development and utilization of MIH as a feed ingredient remains largely unexplored. It was hypothesized that, in the in vitro experiment, different substitution levels of MIH would influence fermentation traits and gas production due to their potential feeding value and bioactive materials. Therefore, the present study aims to verify the application value of MIH as a substitute for corn in the rumen fermentation of goats using in vitro fermentation technology.

## 2. Materials and Methods

The entire experimental process, including the in vitro fermentation experiment, subsequent data processing, and statistical analysis, was conducted at the Zhanjiang Experimental Station (ZES) of the Chinese Academy of Tropical Agricultural Sciences (CATAS). All experimental procedures involving donor management in this study were approved by the ZES, CATAS of the Animal Ethics Committee on 6 January 2026 (Approval No.: CATAS-20260002ZES).

### 2.1. Experimental Materials

The MIH was collected from the Germplasm Resource Garden of the Institute of Subtropical Crops, CATAS (21°09′52″ N, 110°16′24″ E). The kernels and husks were separated by mechanical peeling. The husks were dried in an oven at 65 °C to a constant weight, thoroughly ground using a pulverizer, and then passed through a 1 mm sieve. The dry matter (DM), crude protein (CP), organic matter (OM), ether extract (EE), neutral detergent fiber (NDF), and acid detergent fiber (ADF) of the MIH were 93.7%, 8.14%, 89.9%, 8.53%, 41.3% and 29.6%, respectively, as reported by our previous study [21]. The four substrates used MIH to replace corn at 0% (MIH0 group), 5% (MIH5 group), 10% (MIH10 group), and 15% (MIH15 group; Table 1). About 0.4 g of substrate was weighed and placed into a nylon bag along with 9.0 g of glass beads. The bag was sealed with a heat sealer and stored at 4 °C for subsequent experiments.

### 2.2. Rumen Fluid Donors and Their Management

The rumen fluid donors were Leizhou goats, which were fed on the Grass-Livestock Integration Experimental Base of ZES, CATAS. Prior to the experiment, six healthy goats of similar body weight (15.38 ± 0.50 kg) were selected. The donors were fed a total mixed ration (TMR, concentrate-to-roughage ratio at 4:6) and had free access to feed, fresh water and mineral supplements, maintaining stable physiological status in accordance with the experimental requirements.

### 2.3. Experimental Design, In Vitro Procedure and Sample Collection

Before the morning feeding of the experiment day, rumen fluid was collected from the donors, with a volume of 250–300 mL per goat. After collection, the rumen fluid was thoroughly homogenized, aliquoted into sterile plastic bottles, and immediately placed in a constant-temperature water bath at 39 ℃ to preserve its composition and biological activity.

Upon transportation to the laboratory, the rumen fluid was filtered through four layers of gauze to remove impurities. The filtered rumen fluid was transferred into clean beakers pre-loaded with a fixed volume of prepared artificial rumen fluid and mixed at a ratio of 1:2 (rumen fluid: artificial buffer fluid) according to McDougall. The CO_2_ was continuously bubbled into the beakers at 39 °C to maintain an anaerobic environment, ensuring the physiological activity and component stability of the rumen fluid.

A total of 70 Menke fermenters (Model Fortuna, Haberle Labortechnik, Lonsee, Germany) were used per run, which consist of 4 Menke fermenters for 4 treatments at 6 h, 12 h, 24 h, and 48 h incubation times and 3 Menke fermenters for blank and 3 Menke fermenters for standard. The experiment was conducted in 2 runs. A total of 40 mL buffered rumen fluid was added to each Menke fermenter and then kept in a 39 °C water bath for fermentation.

### 2.4. Sample Collected and Analysis

#### 2.4.1. Gas Production Observed

Gas production was observed after fermentation at 0 h, 3 h, 6 h, 9 h, 12 h, 24 h, and 48 h incubation times. When the cumulative gas volume exceeded 100 mL, the gas in the jars was vented to prevent excessive internal pressure.

#### 2.4.2. Chemical Composition for Substrate and Residue Analysis

To terminate the nutrient fermentation at 6 h, 12 h, 24 h, and 48 h, the nylon bags containing fermentation substrate residues were immediately retrieved and placed in ice water (0 °C).

According to AOAC standard methods [22], the DM (method 922.06), CP (method 984.13), and EE (method 920.39) of the substrate were determined. The determination of OM was quantified by extending method 967.05. The analysis of NDF and ADF followed the methods [23,24]. The substrate residual was measured for DM, NDF, and ADF according to the method described above. The degradation of DM, NDF, and ADF was calculated with the initial contents of the 4 substrates at 0 h and after 6, 12, 24, and 48 h of incubation time.

#### 2.4.3. Fermentation Fluid pH and Fermentation Parameters Measure

Fermentation fluid from each Menke fermenter was collected at 48 h post-fermentation and aliquoted into clean centrifuge tubes. An aliquot of 4 mL fermentation fluid was mixed with an equal volume of deproteinization solution (100 g/L metaphosphoric acid and 0.6 g/L croconic acid) before being stored in liquid nitrogen for subsequent rumen fermentation index analysis. In addition, the residue fermentation fluid was collected to measure the rumen flora.

The pH meter was calibrated using a standard buffer solution (pH 7.00) to ensure accurate pH determination. The pH of fermentation fluid in each incubation tube was determined at each preset time point. After pH measurement, the rumen fluid in each tube was aliquoted into sterile centrifuge tubes and clearly labeled with group and time point information.

Volatile fatty acids (VFAs) were determined using gas chromatography (GC) [25,26]. The concentration of ammonia-N and MCP values in fermentation fluid at the 48 h incubation time point were analyzed using a microplate reader following the chemical methods [27,28].

#### 2.4.4. Microbial Distribution

The DNA of the fermentation fluid samples was extracted using an E.Z.N.A. Bacterial DNA Extraction Kit (Omega Bio-Tek, Inc., Norcross, GA, USA) following the manufacturer’s standard protocols strictly. DNA integrity was assessed through 1.0% agarose gel electrophoresis; clear bands without obvious smearing indicated that the extracted DNA remained intact and undegraded. A NanoDrop 2000 spectrophotometer (Thermo Fisher Scientific, Waltham, MA, USA) was used to measure DNA purity and concentration. Qualified DNA samples were hermetically stored at −80 °C to prevent degradation.

The 1492R (5′-GGTTACCTTGTTACGACTT-3′) and 27F (5′-AGRGTTYGATYMTGGCTCAG-3′) were referenced from the classic literature [29] and synthesized by Sangon Biotech (Shanghai, China) with HPLC-verified purity ≥ 99%. These primers specifically amplify the full-length fragment of the bacterial 16S rRNA gene (approximately 1500 bp). The 20 μL PCR reaction system contained 10 μL 2 × Pro Taq Premix, 0.8 μL of each primer (5 μM), 1 μL template DNA (10 ng/μL), and nuclease-free water to adjust the final volume to 20 μL. The PCR thermal cycling program was as the methods described by Cai et al. [20], with temporary storage at 10 °C until subsequent processing.

The products were purified using the PCR Clean-Up Kit (Yuhua Biotechnology, Shanghai, China) to remove impurities such as residual primers, dNTPs, and polymerases. Purified amplicons were accurately quantified using a Qubit 4.0 Fluorometer (Thermo Fisher Scientific, Waltham, MA, USA) and pooled at equimolar concentrations.

Raw sequencing data were generated using the Illumina NextSeq 2000 system (Illumina, Inc., San Diego, CA, USA) with a 2 × 301 bp paired-end protocol at Majorbio Bio-pharm Technology Co., Ltd. (Shanghai, China). The resulting sequence reads were subsequently deposited onto the Majorbio Cloud Platform for bioinformatics analysis (accessed on 7 April 2026). The bioinformatics analysis included quality filtering of raw reads, operational taxonomic unit (OTU) clustering at 97% sequence similarity, taxonomic annotation against the Silva database, and α and β diversity analyses. Additionally, quantitative real-time PCR (qPCR) was used for absolute quantification of key functional microbial groups, and microscopic observation was conducted to analyze spatial distribution characteristics, thereby achieving multi-dimensional characterization of the rumen microbial community.

### 2.5. Statistical Analysis of Experimental Data

The data of gas production, DM, NDF, and ADF degradability, and fermentation parameters were analyzed using SPSS 22.0 software. One-way analysis of variance (ANOVA) was performed for significance testing through the following models:

Y = μ + MIH + Run+ E,

where Y means dependent variable; μ means the overall mean (n = 8); MIH means the MIH levels replacing corn; Run means the effect of experimental run; and E means the residual error. Orthogonal polynomial contrasts were applied to evaluate the linear and quadratic effects of different groups on the measured parameters. Data were presented as means and standard errors. A threshold of *p* < 0.05 was used to determine statistical differences, whereas a tendency toward significance was considered when 0.05 ≤ *p* < 0.10.

Rumen microbial diversity was carried out with the Majorbio Cloud Platform (https://www.majorbio.com). Raw sequencing reads were filtered, removed, and normalized, and representative sequences for each sample were used according to the Silva database. A Venn diagram was constructed to exhibit the overlap and unique operational taxonomic units (OTUs) among different treatment groups, so as to clarify the similarity and difference in microbial community composition. Alpha diversity, including the Chao index and Shannon index, was calculated using the mothur software (http://www.mothur.org/wiki/Calculators (accessed on 10 April 2026)), and the Kruskal–Wallis test was used to analyze intergroup differences in alpha diversity. We performed a Principal Coordinate Analysis (PCoA) based on Bray–Curtis distance to assess the similarity of microbial communities across samples and combined this with the non-parametric PERMANOVA test to determine the statistical significance of differences in microbial community structure between sample groups. LEfSe analysis (http://huttenhower.sph.harvard.edu/LEfSe (accessed on 17 April 2026)) was performed using the criteria LDA > 3 and *p* < 0.05 to identify bacterial taxa with significant differences in abundance at the phylum and genus levels across different groups, thereby clarifying the distinguishing characteristics of microbial communities between groups. The significant marker for all data in this study was defined at *p* < 0.05 and *p* < 0.01 levels.

## 3. Results

### 3.1. Effects of Substitution of Corn with Macadamia integrifolia Husk on In Vitro Gas Production

As shown in Table 2, gas production decreased linearly (*p* <0.05) as corn was replaced by increasing levels of *Macadamia integrifolia* husk.

### 3.2. Effects of Substitution of Corn with Macadamia integrifolia Husk on DM, NDF and ADF Degradability

There was no difference in DMD at 6 h and 12 h incubation among the four groups (*p* > 0.05; Table 3). However, the DMD decreased linearly at the 24 h and 48 h incubation time points as corn was replaced by increasing levels of MIH (*p* < 0.01). The NDFD decreased linearly at the 6 h, 12 h, 24 h, and 48 h incubation time points as corn was replaced by increasing levels of MIH (*p* < 0.01). The ADFD was no different at the 6 h, 12 h, 24 h, and 48 h incubation time points among the four groups (*p* > 0.05).

### 3.3. Effects of Substitution of Corn with Macadamia integrifolia Husk on In Vitro Fermentation Parameters

As shown in Table 4, the pH of fermentation broth in each group differed highly significantly among the treatment groups at 48 h (*p* < 0.001), with a statistically significant quadratic effect (*p* < 0.05). Specifically, pH decreased and then increased with the increasing substitution ratio of MIH levels. The pH of the MIH5 group decreased slightly compared with the MIH0 group, while that of the MIH10 and MIH15 groups increased compared with the MIH0 group. There were no differences in TVFAs, isobutyric acid, and valeric acid concentration among the four experimental groups (*p* > 0.05). Among them, the contents of ammonia nitrogen, butyric acid, and isovaleric acid were the highest in the control group; microbial crude protein (MCP), acetic acid, and propionic acid were the highest in the MIH5 group; and the acetate/propionate ratio (A/*p* ratio) was the highest in the MIH15 group.

### 3.4. Bacterial Community Composition in In Vitro Fermentation at 48 H

**Figure 1:**
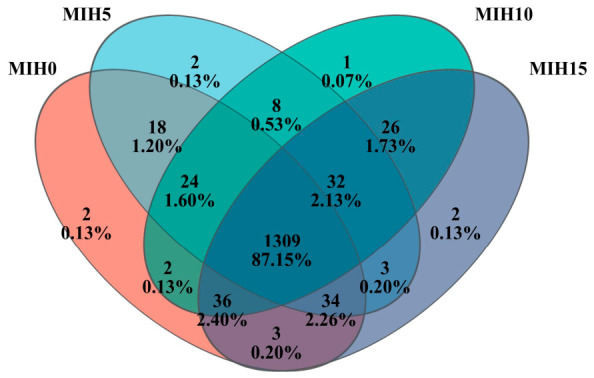
Distribution of microbial OTUs in different treatment groups. Note: MIH0 = MIH replaced corn at 0%; MIH5 = MIH replaced corn at 5%; MIH10 = MIH replaced corn at 10%; MIH15 = MIH replaced corn at 15%; n = 8 per treatment.

**Figure 2:**
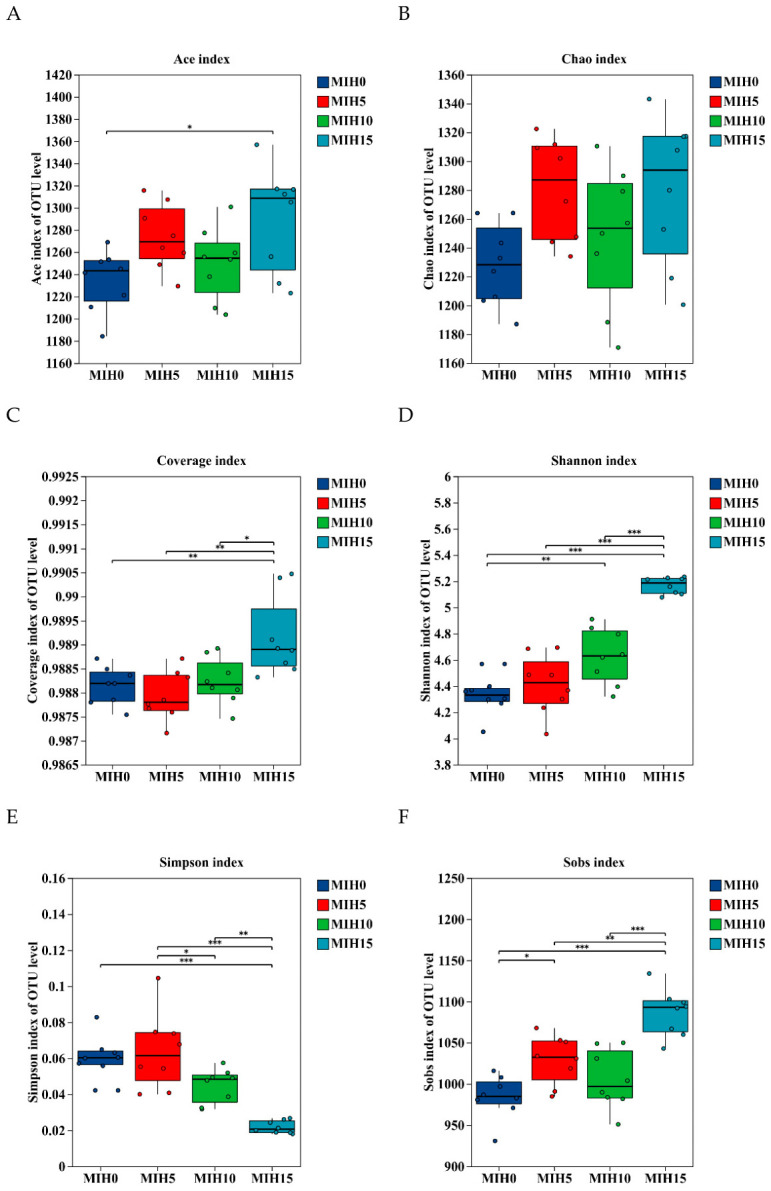
Effects of substitution of corn with MIH on α-diversity indices of in vitro fermentation microorganisms in goats. (**A**), ace index; (**B**) Chao index; (**C**) Coverage index; (**D**) Shannon index; (**E**) Simpson index; (**F**) Sobs index. Note: * shows difference at *p* < 0.05, ** shows difference at *p* < 0.01, *** shows difference at *p* < 0.001. MIH0 = MIH replaced corn at 0%; MIH5 = MIH replaced corn at 5%; MIH10 = MIH replaced corn at 10%; MIH15 = MIH replaced corn at 15%; n = 8 per treatment. ANOVA = effect of MIH replaced corn at different levels; linear = linear effect of MIH replaced corn at different levels; quadratic = quadratic effect of MIH replaced corn at different levels.

In total, 1502 OTUs were obtained from 32 samples in the experiment (Figure 1). The four groups shared 1309 OTUs. The number of unique OTUs in the MIH0, MIH5, MIH10, and MIH15 groups was 2, 2, 1, and 2, respectively. The Chao index was not different among the four groups (*p* > 0.05; Figure 2B). The Ace index differed significantly between the MIH0 and MIH15 groups (*p* < 0.05; Figure 2A). The Shannon, Simpson, and Sobs indices differed significantly among groups (*p* < 0.05; Figure 2D–F). The Coverage index of all samples was ≥0.98, indicating sufficient sequencing depth (Figure 2C).

**Figure 3:**
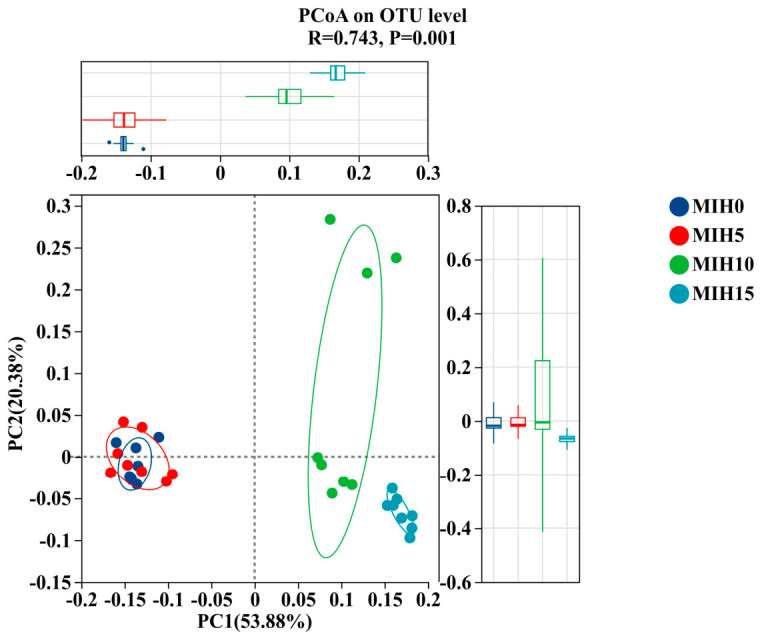
PCoA analysis of rumen bacterial community at the OTU level and differences in β-diversity indices. Note: MIH0 = MIH replaced corn at 0%; MIH5 = MIH replaced corn at 5%; MIH10 = MIH replaced corn at 10%; MIH15 = MIH replaced corn at 15%; n = 8 per treatment. ANOVA= effect of MIH replaced corn at different levels; linear = linear effect of MIH replaced corn at different levels; quadratic = quadratic effect of MIH replaced corn at different levels.

PCoA analysis was performed based on the Bray–Curtis distance (Figure 3). The differences among the four groups were significant (*p* < 0.05), and the inter-group difference was significantly larger than the intra-group difference (R = 0.743), indicating valid experimental grouping. The experimental treatment also had a significant driving effect on the bacterial community structure. The box plot shows the score distribution of the four groups on the PC1 axis. The scores of MIH0 and MIH5 were concentrated on the left, while those of MIH10 and MIH15 were biased to the right, and the sample points of the MIH10 group were scattered. PC1 and PC2 explained 53.88% and 20.38% of the total variation, respectively, with a cumulative contribution of 74.26%, reasonably reflecting the similarity differences in rumen bacterial community structure among the four treatment groups.

**Figure 4:**
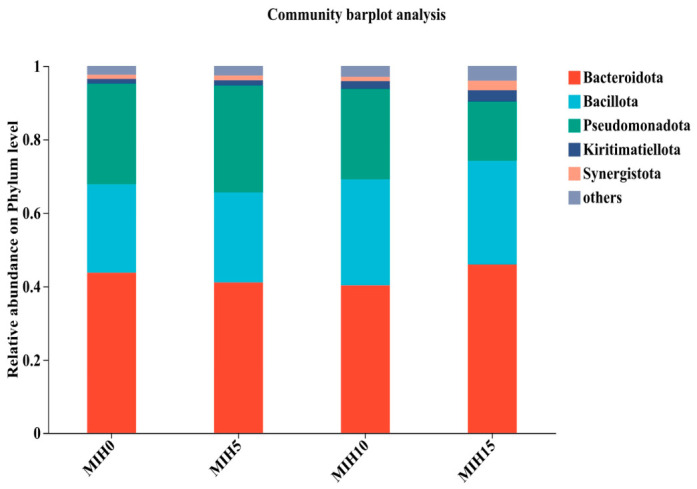
Relative abundance in the fermentation fluid at the phylum level among the four groups.

Species annotation of the four sample groups identified a total of 27 bacterial phyla (Figure 4). The Bacteroidota phylum was the dominant phylum with the highest relative abundance across all treatment groups, with relative abundances of 43.74%, 41.07%, 40.29%, and 45.98% in the MIH0, MIH5, MIH10, and MIH15 groups, respectively. The Pseudomonadota phylum was the second most dominant phylum in the MIH0 and MIH5 groups, with relative abundances of 27.35% and 29.06%, respectively; the Bacillota phylum was the second most dominant phylum in the MIH10 and MIH15 groups, with relative abundances of 28.84% and 28.20%, respectively.

**Figure 5:**
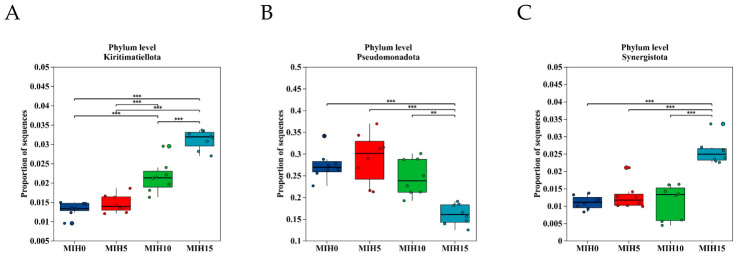
Significant difference at the phylum level in fermentation fluid samples at 48 h. (**A**) Kiritimatiellota phylum; (**B**) Pseudomonadota; (**C**) Synergistota. Note: ** shows difference at *p* < 0.01, *** shows difference at *p* < 0.001. MIH0 = MIH replaced corn at 0%; MIH5 = MIH replaced corn at 5%; MIH10 = MIH replaced corn at 10%; MIH15 = MIH replaced corn at 15%; n = 8 per treatment. ANOVA = effect of MIH replaced corn at different levels; linear = linear effect of MIH replaced corn at different levels; quadratic = quadratic effect of MIH replaced corn at different levels.

There were significant differences in the abundances of each phylum across the different treatment groups (Figure 5), the relative abundance of the Kiritimatiellota phylum was highest in the MIH15 group and lowest in the MIH0 group; Pseudomonadota had the highest abundance in the MIH5 group and the lowest in the MIH15 group; Synergistota similarly exhibited the highest abundance in the MIH15 group and the lowest in the MIH0 group.

**Figure 6:**
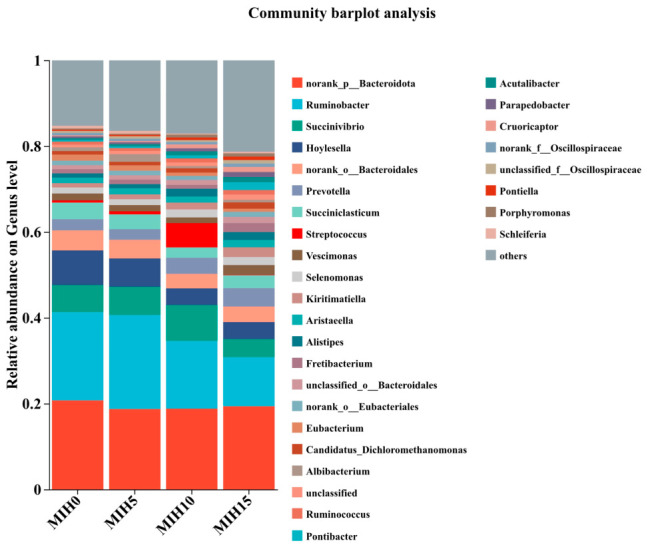
Relative abundance in the fermentation fluid at the genus level among the four groups.

A total of 326 genera were identified among the four groups (Figure 6). The genus *norank-p-Bacteroidota* was the dominant genus with the highest relative abundance in the MIH0 and MIH10 groups, with relative abundances of 20.72% and 18.79%, respectively. The genus *Ruminobacter* was the second most dominant genus in the MIH0 group, with a relative abundance of 20.59%; the genus Other was the second most dominant genus in the MIH10 group, with a relative abundance of 16.73%. The genus *Ruminobacter* was the most dominant genus in the MIH5 group, with a relative abundance of 21.90%, while the “Other” genus was the dominant genus with the highest relative abundance in the MIH15 group, at 20.52%. The *norank-p-Bacteroidota* genus ranked second in both the MIH5 and MIH15 groups, with relative abundances of 18.71% and 19.37%, respectively.

**Figure 7:**
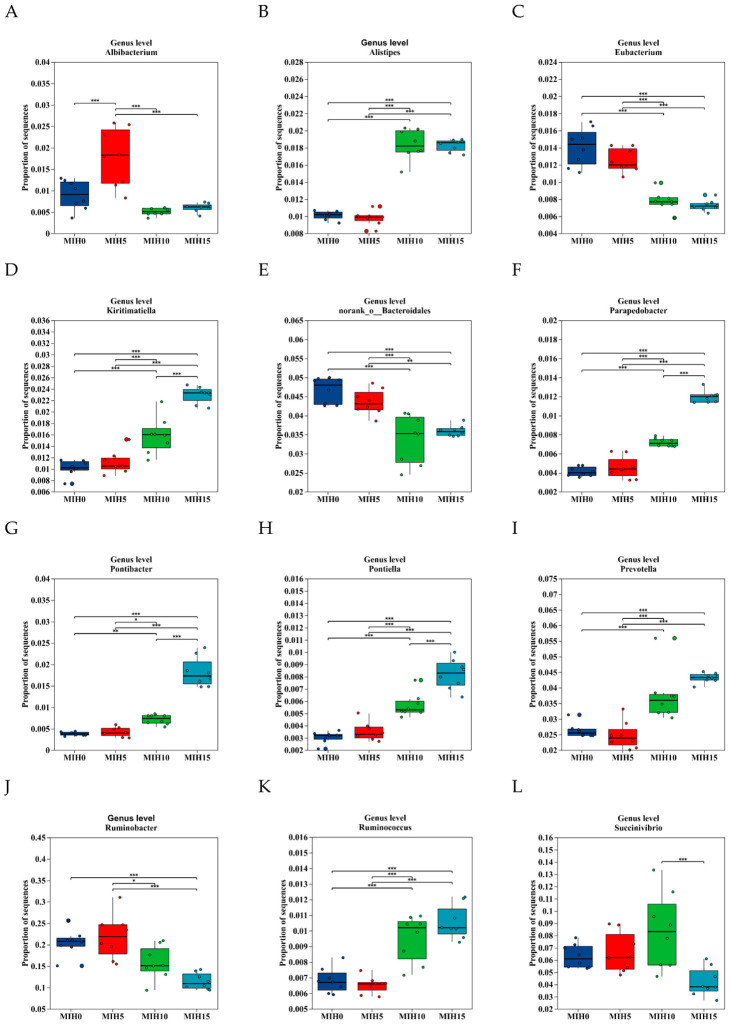
Significant difference at the genus level in fermentation fluid samples at 48 h. (**A**) *Albibacterium*; (**B**) *Alistipes*; (**C**) *Eubacterium*; (**D**) *Kiritimatiella*; (**E**) *norank-o-Bacteroidales*; (**F**) *Parapedobacter*; (**G**) *Pontibacter*; (**H**) *Pontiella*; (**I**) *Prevotella*; (**J**) *Ruminobacter*; (**K**) *Ruminococcus*; (**L**) *Succinivbrio*. Note: * shows difference at *p* < 0.05, ** shows difference at *p* < 0.01, *** shows difference at *p* < 0.001. MIH0 = MIH replaced corn at 0%; MIH5 = MIH replaced corn at 5%; IH10 = MIH replaced corn at 10%; MIH15 = MIH replaced corn at 15%; n = 8 per treatment. ANOVA = effect of MIH replaced corn at different levels; linear = linear effect of MIH replaced corn at different levels; quadratic = quadratic effect of MIH replaced corn at different levels.

At the genus level, the relative abundances of the 12 dominant genera identified in this study showed significant differences across the different treatment groups (*p* < 0.05; Figure 7A–L). The relative abundance of *Albibacterium* was higher in the MIH5 group than in the MIH0 group (*p* < 0.001); while *Ruminobacter*, although most abundant in the MIH5 group, showed only a slight increase compared to the MIH0 group. Overall, the addition of a moderate dose of MIH significantly promoted the enrichment of *Albibacterium*, while it had only a slight enhancing effect on *Ruminobacter*. In addition, the relative abundances of *Eubacterium* and *norank-o-Bacteroidales* were significantly higher in the MIH5 group than in the MIH10 and MIH15 groups; there was no difference between the MIH0 and MIH5 groups, and no difference between the MIH10 and MIH15 groups.

Further analysis of the differences in bacterial genus abundance revealed that the bacterial genera significantly enriched in the medium- and high-dose treatment groups can be classified into two distinct patterns of change. The first group includes *Alistipes*, *Prevotella*, and *Ruminococcus*, whose relative abundances were significantly higher in the MIH10 and MIH15 groups than in the MIH0 and MIH5 groups, but there was no significant difference between the MIH10 and MIH15 groups. The second group, represented by *Kiritimatiella*, *Parapedobacter*, *Pontibacter*, and *Pontiella*, also showed significantly higher relative abundances in the MIH10 and MIH15 groups compared to the MIH0 and MIH5 groups. Furthermore, their abundances increased with higher doses of MIH, with the MIH15 group showing significantly higher levels than the MIH10 group.

**Figure 8:**
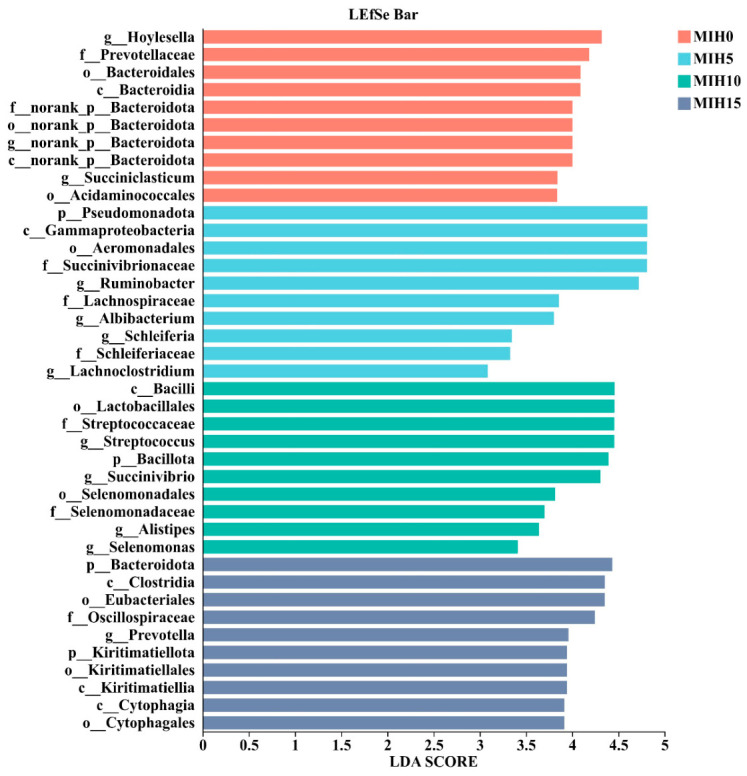
LDA discriminant plot of differential genera based on LEfSe analysis. Note: MIH0 = MIH replaced corn at 0%; MIH5 = MIH replaced corn at 5%; MIH10 = MIH replaced corn at 10%; MIH15 = MIH replaced corn at 15%; n = 8 per treatment. ANOVA = effect of MIH replaced corn at different levels; linear = linear effect of MIH replaced corn at different levels; quadratic = quadratic effect of MIH replaced corn at different levels.

LEfSe analysis (LDA-value > 3.0, *p* < 0.05) was performed on the four groups of samples, and a total of 280 differentially enriched microbial biomarkers were screened among the groups. The effect size distribution is shown in Figure 8. The MIH0, MIH5, MIH10, and MIH15 groups had 10, 10, 10, and 10 biomarkers, respectively. The core differential species of the MIH5 group was p__Pseudomonadota (LDA = 4.82), and the overall enrichment effect of the MIH5 group was stronger than that of the other treatment groups. The characteristic flora of the MIH10 group was dominated by p__Bacillota, with LDA values ranging from 4.39 to 4.44. The differential taxa of the MIH15 group included p__Bacteroidota (LDA = 4.47). The LDA values of all differential biomarkers ranged from 3.02 to 4.82.

## 4. Discussion

### 4.1. Effects of Substitution of Corn with Macadamia integrifolia Husk on Gas Production and Nutrient Digestibility

In vitro gas production is highly correlated with the metabolizable energy (ME) content of feeds, and gas production kinetics can be used to estimate ME [30,31]. In general, greater substrate degradation and more complete energy release result in higher ME values. In the present study, gas production in all treatment groups gradually increased with fermentation time, which is consistent with findings from substitution experiments using total mixed rations for dairy cows [32] and high-protein agro-industrial co-products [33]. Similarly, Jolazadeh and Azizi [34] reported that gas production from camellia seed pods (CSP) increased significantly throughout the incubation period and was higher than that from wheat straw at several time points. The gradual, rather than rapid, increase in gas production observed in this experiment may be attributed to: (i) the low content of rapidly fermentable non-fibrous carbohydrates (NFC) in *Macadamia integrifolia* husk [8,35]; (ii) the tight binding among cellulose, hemicellulose, and lignin [36]; and (iii) the inherent difficulty of microbial cell wall degradation [37]. In addition, Zhang et al. reported that substituting corn with different levels of dried persimmon peel could reduce the gas production in Guanzhong dairy goats [18], which is in agreement with our results. This could be explained by a decline in DMD. During fermentation, the degradabilities of DM, NDF, and ADF all improved to some extent. This pattern aligns with the results of Zhang et al., who studied nutrient utilization in sheep rumen fluid using different proportions of stevia stalks [38]. Similarly, Hoffman et al. demonstrated that DM and NDF degradabilities increased with longer rumen culture time in perennial forages at three growth stages [39]. Furthermore, Van Soest et al. confirmed that ADF, as a structural fiber, is also degraded by rumen microorganisms, resulting in a slow and gradual increase in its degradability [40]. Related studies have confirmed that the synergistic modification of straw using a lactic acid bacteria-cellulase complex can effectively cleave the bonds between cellulose, hemicellulose, and lignin; improve the ruminal degradability of the substrate; and significantly increase the apparent digestibility of ADF [41]. Moreover, our results showed that the degradabilities of DM, NDF, and ADF decreased with increasing MIH levels, which could partly be explained by the core having a greater NFC than MIH.

### 4.2. Effects of Substitution of Corn with Macadamia integrifolia Husk on In Vitro Fermentation Parameters

According to the optimal range of rumen fermentation proposed by Van Soest [42], the optimal pH for the normal growth, metabolism, and fiber degradation of fibrolytic bacteria is 6.2–6.8. In this study, the in vitro fermentation conditions of each group were stable and suitable, and the rumen fluid pH of each treatment group was maintained between 6.30 and 6.36. It can be seen that the pH of each group under the experimental conditions was within the appropriate range, indicating that replacing corn with MIH did not destroy the acid-base balance in the rumen and the fermentation of the internal environment remained stable. In addition, this result is in agreement with the experiment by researchers on replacing alfalfa hay with pistachio hulls in sheep diets [43]. The anaerobic environment of the rumen led to incomplete substrate degradation, and the main products of fermentation were volatile fatty acids (mainly acetic acid, propionic acid, and butyric acid), carbon dioxide, and methane [44,45]. The MIH are rich in monounsaturated fatty acids, such as oleic acid and palmitoleic acid, with conservative species-specific compositions [46]. Moreover, the MIH contains a certain amount of unsaturated fatty acids (UFA) [47]. During rumen digestion, such substances affect the activity of fibrolytic bacteria and microbial community structure, change the proportion of related volatile fatty acids, and affect the synthesis efficiency of MCP. Gao et al. found that the effect of unsaturated fatty acids on MCP varies with the degree of unsaturation, and adding 3% long-chain unsaturated fatty acids can reduce protozoal protein content [48]. The study also reported that MCP is mainly a protozoal protein, so unsaturated fatty acids have a protozoal protein inhibition pathway, reducing the synthesis efficiency of total MCP.

Urea enters the rumen and is rapidly hydrolyzed to ammonia nitrogen under the action of urease, which is an important nitrogen source for rumen bacteria [48]. Previous studies confirmed that urea is a very important nitrogen source [49]. In addition, the optimal ruminal NH_3_-N concentration for maximal total microbial crude protein (MCP) synthesis is 5–8 mg/100 mL [48]. In the present study, the NH_3_-N concentration in all treatment groups exceeded 5 mg/100 mL, indicating that the ammonia supply had met the microbial demand. Under this condition, MCP synthesis reached a plateau and did not increase further with rising NH_3_-N concentration. Interestingly, among them, the ammonia nitrogen of the MIH5 group was 7.69 mg/100 mL, which was within the optimal range of 5–8 mg/100 mL, and the MCP content reached the highest, further indicating that the synthesis efficiency of microbial protein is optimal when ammonia nitrogen is within the appropriate range. This experiment indirectly confirmed the accuracy of the optimal rumen ammonia nitrogen concentration theory [50].

Based on thermogravimetric analysis (TGA/DTG), the MIH contains 35% cellulose, 30% hemicellulose, and 35% lignin, which is similar to that of cereal straws [51,52]. In the present study, increasing the dietary substitution level of MIH progressively shifted the overall nutritional profile toward high fiber content. According to Jo et al. [53], the composition and nutritional profile of diets are typically associated with increased propionic acid and butyric acid concentrations, as well as a decreased acetate:propionate ratio. It was reported that substitution of 20% of corn with dried persimmon peel could improve the TVFA, acetic acid, isobutyric acid, butyric acid, and valeric acid concentrations in an in vitro study [18]. Higher molar proportions of acetic acid were found in almond or hazelnut replacement of corn at 14 and 21 level groups, while the iso-VFAs were reduced by hazelnut skin groups regardless of the dose replacement of corn. The proportion of butyric acid showed lower values in almond replacement of corn at the 21 level group and hazelnut replacement of corn at the 14 level group compared to the control group [16]. In contrast, in the present study, we found that the total concentrations of acetic acid, propionic acid, isobutyric acid, butyric acid, isovaleric acid, and valeric acid decreased when the corn was replaced by MIH. This could partly be explained by the fact that MIH has higher fiber content and less non-fiber carbohydrate content than corn.

### 4.3. Effects of Substitution of Corn with Macadamia integrifolia Husk on In Vitro Microbial Flora

Rumen hosts a diverse array and abundance of bacteria, which is regarded as a specialized digestive system in ruminants to digest and ferment. This study elucidated the divergent modulation patterns of substitution of corn with MIH in goat rumen microbiota through alpha and beta diversity analyses. A previous study reported a greater alpha diversity index, hinting at a potential trend towards a more diverse and abundant rumen microbiota [54]. In the present study, we found that a part of the alpha indices, for example, Ace, Shannon, coverage, and Sobs indices, were increased with MIH replacing the substrate corn. Beta diversity analysis revealed distinct structural shifts across treatment groups, which suggests the MIH supplementation reshaped microbial interaction networks.

Generally, at the phylum level, Bacteroidetes and Bacillota were regarded as the predominant bacterial phyla in goats [55], sheep [56], cattle [57], and dairy cows [58]. The Pseudomonadota phylum, formerly known as Proteobacteria, is a major phylum of Gram-negative bacteria with diverse metabolic capabilities, ecological roles, and both pathogenic and beneficial species (using horizontal metaproteomics and CAZymes analysis of lignocellulolytic microbial consortia with selective abundance in the rumen of ruminants and gut of termites). Interestingly, in the present study, we found that the relative abundance of this phylum was numerically greater than that of Bacillota and was the second most dominant phylum in the MIH0 and MIH5 groups. Synergistota plays an important role in the potential to detoxify harmful compounds found in plants grazed by ruminants [59]. Our results showed that the relative abundance of Synergistota was greater in MIH10 and MIH15 groups, which is possibly explained by MIH containing phenolic acid.

Reports on the dominant ruminal bacterial genus are inconsistent and mainly depend on animal species and dietary composition [60]. In the present study, we found that the dominant genera are *norank_p-Bacteroidota* and *Ruminobacter*, which is partly in agreement with a previous in vitro study in Leizhou goats [21]. The NDF and ADF of the four substrates increased when MIH replaced corn. In addition, the NDFD was greater for the MIH0 group than for the other three groups. That is, the substrate residues were lowest in the MIH0 group. In the present study, a part of the cellulose-decomposing bacteria, including *Alistipes* [61] and *Ruminococcus* [62], was lower in the MIH0 and MIH5 groups and higher in the MIH10 and MIH15 groups. This suggests that MIH could replace 5% of corn, whereas 10% and 15% could decrease the relative abundance of cellulose-decomposing bacteria. If necessary, a suitable dose of cellulases and pectinases or probiotics could be added when using MIH to replace 10% or 15% of corn. A part of amylolytic bacteria, including *Ruminobacter* [63] and *Succinivibrio* [64], was lowest in the MIH15 group, which could be explained by the decreased concentration of starch in the substrate.

## 5. Conclusions

The effects of the substitution of corn with MIH were investigated using an in vitro rumen fermentation system. The degradation rates of DM, NDF, and ADF decreased with the increasing MIH levels; the MIH5 group had the highest concentrations of MCP, TVFAs, acetic acid, and propionic acid among the four groups. These results indicate that MIH can serve as a sustainable alternative to corn in goat diets. We suggest MIH replace 5% corn as the optimal dosage in the diet of goats. Unfortunately, this study employed an in vitro batch culture model, which cannot reflect growth performance and physiological metabolic conditions in goats. Hence, more in vivo studies need to focus on growth performance, serum metabolism, and energy and nitrogen balance when using MIH as feed stuff.

## Figures and Tables

**Table 1:** Ingredient composition and nutrition level of the four substrates.

Ingredients	MIH0	MIH5	MIH10	MIH15
Peanut vine	40.00	40.00	40.00	40.00
*Macadamia integrifolia* husk, ground	0.00	5.00	10.00	15.00
Corn	30.00	25.00	20.00	15.00
Soybean meal	12.00	11.20	10.90	11.00
Wheat bran	4.30	8.60	10.70	11.00
Soybean hulls	9.60	5.90	3.60	2.40
Soybean oil	0.10	0.30	0.80	1.60
Premix ^1^	4.00	4.00	4.00	4.00
Nutritional levels	-	-	-	-
Metabolizable energy, MJ/kg ^2^	9.54	9.53	9.52	9.54
Digestible energy, MJ/kg ^2^	11.62	11.60	11.60	11.62
Dry matter, %	85.20	85.28	85.16	84.81
Crude protein, %	13.01	13.02	13.03	13.03
Ether extract, %	2.62	2.94	3.19	3.41
Neutral detergent fiber, %	36.59	36.95	37.62	38.52
Acid detergent fiber, %	23.99	24.06	24.55	25.37

^1^ The per kg of premix provided: VA 4000 IU, VD3 1600 IU, VE 36 mg, I 0.12 mg, Cu 10 mg, Fe 50 mg, Mn 42 mg, Zn 40 mg, and Se 0.12 mg. ^2^ Calculated values. Note: MIH0 = MIH replaced corn at 0%; MIH5 = MIH replaced corn at 5%; MIH10 = MIH replaced corn at 10%; MIH15 = MIH replaced corn at 15%.

**Table 2:** Effects of substitution of corn with *Macadamia integrifolia* husk on in vitro gas production.

Items	Time/h	Different Treatment Groups				SEM	*p*-Value		
		MIH0	MIH5	MIH10	MIH15		ANOVA	Linear	Quadratic
Gas production	3	7.61	8.66	7.25	7.30	0.22	0.050	0.193	0.218
	6	17.17	16.89	15.60	15.36	0.28	0.044	0.007	0.968
	9	25.69	25.26	23.27	22.84	0.41	0.018	0.003	1.000
	12	31.84	31.89	29.57	28.00	0.56	0.023	0.004	0.422
	24	47.89	47.15	44.62	41.39	0.91	0.036	0.006	0.451
	48	66.76	68.56	60.92	54.93	1.67	0.007	0.002	0.172

Note: MIH0 = MIH replaced corn at 0%; MIH5 = MIH replaced corn at 5%; MIH10 = MIH replaced corn at 10%; MIH15 = MIH replaced corn at 15%; n = 8 per treatment. ANOVA = effect of MIH replaced corn at different levels; linear = linear effect of MIH replaced corn at different levels; quadratic = quadratic effect of MIH replaced corn at different levels.

**Table 3:** Effects of substitution of corn with *Macadamia integrifolia* husk on DM, NDF, and ADF degradability.

Items	Time/h	Different Treatment Groups				SEM	*p*-Value		
		MIH0	MIH5	MIH10	MIH15		ANOVA	Linear	Quadratic
Dry matter digestibility, (DMD), %	6	24.93	26.08	24.45	24.27	0.563	0.700	0.487	0.571
	12	30.79	34.55	29.59	29.34	0.838	0.097	0.197	0.215
	24	37.71	36.33	34.64	33.13	0.496	0.003	<0.001	0.929
	48	51.46	48.81	43.83	40.98	1.165	0.033	<0.001	0.960
Neutral detergent fiber digestibility (NDFD), %	6	14.44	12.04	10.78	8.80	0.851	0.189	0.049	0.905
	12	18.76	19.05	16.45	11.92	0.727	0.031	0.005	0.111
	24	24.27	21.10	20.09	17.84	0.703	0.013	0.003	0.708
	48	33.02	33.20	28.47	25.05	1.066	0.012	0.002	0.318
Acid detergent fiber digestibility (ADFD), %	6	11.01	10.41	10.51	8.34	0.627	0.559	0.201	0.578
	12	13.84	12.99	11.02	10.47	0.940	0.607	0.220	0.942
	24	16.60	15.01	14.01	13.70	1.471	0.908	0.494	0.838
	48	24.70	24.97	23.11	22.67	1.471	0.942	0.606	0.915

Note: MIH0 = MIH replaced corn at 0%; MIH5 = MIH replaced corn at 5%; MIH10 = MIH replaced corn at 10%; MIH15 = MIH replaced corn at 15%; n = 8 per treatment. ANOVA = effect of MIH replaced corn at different levels; linear = linear effect of MIH replaced corn at different levels; quadratic = quadratic effect of MIH replaced corn at different levels.

**Table 4:** Effects of substitution of corn with *Macadamia integrifolia* husk on in vitro fermentation parameters.

Items	Different Treatment Groups				SEM	*p*-Value		
	MIH0	MIH5	MIH10	MIH15		ANOVA	Linear	Quadratic
pH	6.31	6.30	6.33	6.36	0.005	<0.001	<0.001	0.020
Ammonia-N, mg/100 mL	9.85	7.69	8.48	7.00	0.305	<0.001	<0.001	0.405
MCP, mg/100 mL	10.44	11.07	9.05	9.83	0.215	0.001	0.012	0.822
TVFAs, mmoL/L	34.03	35.05	32.91	32.65	0.453	0.223	0.124	0.472
Acetic acid, mmoL/L	22.06	22.22	20.57	20.52	0.347	0.141	0.047	0.868
Propionic acid, mmoL/L	9.61	9.66	8.81	8.52	0.159	0.008	0.001	0.487
Isobutyric acid, mmoL/L	0.40	0.39	0.36	0.35	0.010	0.257	0.060	0.991
Butyric acid, mmoL/L	2.74	2.39	2.51	2.42	0.049	0.032	0.034	0.141
Isovaleric acid, mmoL/L	0.63	0.59	0.55	0.55	0.013	0.045	0.007	0.442
Valeric acid, mmoL/L	0.32	0.33	0.30	0.30	0.006	0.204	0.124	0.576
Acetate to propionate ratio	2.23	2.32	2.34	2.41	0.018	<0.001	<0.001	0.600

Note: MIH0 = MIH replaced corn at 0%; MIH5 = MIH replaced corn at 5%; MIH10 = MIH replaced corn at 10%; MIH15 = MIH replaced corn at 15%; n = 8 per treatment. ANOVA= effect of MIH replaced corn at different levels; linear = linear effect of MIH replaced corn at different levels; quadratic = quadratic effect of MIH replaced corn at different levels.

## Data Availability

Data will be made available on request.
